# The Ethics of the Dental Profession

**Published:** 1858-03

**Authors:** B. P. Aydelott

**Affiliations:** President of the Board of Trustees


### THE ETHICS OF THE DENTAL PROFESSION.

An Address delivered at the Commencement of the Ohio Dental College, Cincinnati, February 17th, 1858.

BY B. P. AYDELOTT, D. D.,
President of the Board of Trustees.

How each one of us ought to conduct himself, is certainly a most important inquiry. Ignorance, or even uncertainty here, will render every other species of knowledge nearly useless.

Whatever a man's disposition, ability, or attainments may be, and whatever other advantages he may possess, yet, unless he enjoys the confidence of the wise and the good, he can accomplish very little that is desirable for himself or for others. But the confidence of the wise and the good can be gained only by right conduct. With this confidence even a man of quite moderate abilities and slender attainments may achieve much for himself, and be quite useful in the world. He will be respected and happy, and deservedly so. How unspeakably momentous, then, is right conduct to every one, whatever his talents, his place, or his circumstances! Can we wonder, therefore, that not merely the practice, but the study also of such conduct, has occupied the minds of the best men in all ages?

Here, however, let it be premised that, not a small or an unimportant part of human conduct is, and ought to be, regulated purely by the usages of society, and by the ideas and sentiments entertained by the community around us about propriety of deportment and address. But in all this there is, strictly speaking, involved no moral principle whatever. Hence a man may attain to great perfection here, become an almost faultless model of propriety and politeness—a Chesterfield, a Burr—and yet be a very bad man; as the world to its sorrow has often witnessed. And another, also, may be very defective in the graces of easy manners and an elegant address, and yet be really a good man notwithstanding. But it manifestly would not hurt this good man, or render him less useful or lovable to cultivate the manners of a gentleman.

Further, let it here also be particularly observed that what is commonly called politeness or good manners so runs into and interlaces, at some points, with the domain of morals as to become *there* really a part of it. Now, in all such cases, moral law must be supreme. The usages of society must give place to the claims of duty, should these at any time come into conflict. No man can ever be required to sacrifice his conscience to his politeness. But we should be careful here never to make conflicts between morality and politeness, where these do not really exist; or to bring in conscience as a mere excuse for rude manners. To do this is the sure way to impair the influence of conscience, and to render morality unnecessarily repulsive.

But let us, without further preface, proceed to the subject of the evening.

We design, in the following official Address, to direct your attention to the subject of Ethics or Moral Philosophy in its relations to the Dental Profession.

We shall not here consider the principles of moral science and the fruits or practical results of these apart; neither shall we handle either in a systematic manner. The occasion precludes this.

Neither shall we adopt the method followed by some ethical writers, viz: first, to treat of duties towards one's self, and then, secondly, of duties towards others. For this would necessarily lead to so much of repetition that our time would be exhausted before we were half through the subject.

But in order to present within moderate limits a general view of the subject, both in its great principles and the practical applications of these, and that in such a manner, too, as shall be most easily understood and remembered, we propose to consider the conduct becoming the dentist, not in its personal and relative aspects, that is, not what he owes to himself and to others; but in its inherent ethical character, that is, what ought his conduct to be, in itself considered, whoever or whatever be its object. Our theme, then, is—

HOW OUGHT THE DENTIST TO CONDUCT HIMSELF IN THE PRACTICE OF HIS PROFESSION?

We answer—he ought to practice his profession, in the first place,

I. JUSTLY. This, no man who has a conscience, or even a common regard for his own character, will deny. But while the general truth will be admitted by all, there is undoubtedly a great want of information in respect to what justice requires in many particular cases; and there may also be more or less of an honest diversity of sentiment even where there is much intelligence.

Let us, then, consider somewhat particularly what justice seems to require of the dental practitioner. As first—

1. He certainly ought to be *well acquainted with his profession*, both theoretically and practically.

We do not mean that every one engaged in dentistry should be perfectly learned in his science, and a thorough practitioner, and that no other can justly engage in the profession. For if this were the case, none could ever enter upon the practice, and the profession would soon die out. But this we do mean, that no one can justly embark in the practice who has not, in the judgment of the profession itself, passed through such a course of study and such a training in the manipulations of dentistry as will best fit him for beginning a professional career. Only the young dentist thus prepared has a right to regard himself as one competent, safely and beneficially to the public, to practice his profession. He only is duly qualified to make that subsequent self-improvement, also, which he ought; and to contribute somewhat toward the advancement of his science and art, and thus do honor to his profession, and benefit the world.

And as no one, especially in so important a matter, can be a competent judge in his own case, so no one should embark in the practice of dentistry till, like the student of medicine or of law, he has been examined and approved by the constituted guardians of the profession. In other words, he ought to be graduated, or, at least, licensed by those whose authority to teach and commission has been legally or generally recognized. Again,—it is implied in a just conduct that the dentist be—

2. *Faithful in his work.* We have supposed that he comes into the profession well qualified by study and diligent training, to begin the practice. So far, then, he has acted justly; but something more is necessary, if he would retain a character for justice. It is not sufficient that he is competent to begin the practice of the profession, but he must faithfully practice it. This implies not only that he be diligent in study so as to keep up with the progress of the profession, but that he duly examine each particular case as it comes before him; that he give to his patient honestly and frankly the best counsel he can, and that in carrying out the views he has formed, he leaves nothing he can command undone towards success, and that he goes through his whole work—not thoughtlessly, slovenly, or superficially—but to the best of his ability.

He who is not thus faithful in his work is not a just man. He does not deserve the confidence and respect of the public; he injures dental science; he disgraces his profession; he ought not to have an easy conscience. He is a bad man.

Further, the dental practitioner ought to be—

3. *Faithful* in all his engagements. Certainly veracity is an essential element in a just character. But not only the conscious liar and deceiver,—the man also who is *careless of his word* is guilty of injustice. A professional promise is a sacred thing, and ought to be faithfully observed. Nothing but a real necessity can justify any one in not fulfilling his every engagement.

But it is painful to think how lightly some men will make appointments, and how careless they are about the fulfilment of them. The evils resulting from this conduct are innumerable, and often very serious. The loss of time, the suffering and dangers it brings upon the patient, and the injury it inflicts upon the profession—to say nothing of the reputation of the offender—these are some of its evils; it would be impossible to calculate their fearful amount.

Let every practitioner, then, who wishes to maintain a character for justice here, be careful, first, how he makes engagements—let him so calculate his time and business that he can do everything in its season, and yet never be in a hurry so as to be tempted to do it poorly; and then, secondly, when he has made appointments, let him conscientiously use his utmost endeavors to meet them; let him make everything else bend and give place to his professional engagements.

Again, justice requires that the dentist be—

4. *Equitable in his charges.* The true professional spirit would indeed lead a man, so far as he possesses it, to pursue the science of his profession for its own sake, and freely to confer its benefits upon his fellow-men. This is a noble passion, and all its aspirations and influences are elevating. It is this which distinguishes a profession from a trade or a mere money-making calling.

Still, as no one can live in this world without means, and, as a general rule, these means must be gained by each one of us for himself, so the professional man, like others, must be paid for his services. Simple justice demands this. Let it be considered, also, that without these gains, multitudes of the finest minds would be kept out of the professions, and these would very slowly, if ever, attain to perfection. The common usage of the practitioners of every profession in any place has settled what is *there* a just charge for services. And this rule should generally be adhered to.

But there are particular circumstances in many cases which ought to operate, more or less, to reduce such charges, and sometimes do them away altogether. It would not, for example, be equitable to make a laboring man, or a small trader, or a widow in moderate circumstances, pay as much for a costly operation as might rightly be required of a wealthy individual, or a prosperous merchant. Justice for one is not justice for all. Circumstances ought to modify charges. Physicians and lawyers act upon this principle ; and so should dentists.* But we must hasten to another requirement of justice.

5. The conduct of the dentist ought always to be *fair and honorable towards his fellow-practitioners.* Their general reputation, their professional standing, their welfare, and their progress, should be a matter of concern to him. He who is reckless of these proclaims himself a CAIN in his profession. "Am I my brother's keeper?" was the insolent reply of that murderer to his Maker. In a very high and peculiar sense, each professional man is the keeper of his brother. Just as he feels himself so, will he be fair and honorable in all his conduct towards his fellow-practitioners.

Hence, when called into consultation with another, he will

---

* In the case of ministers of the Gospel, the almost universal rule among medical men and lawyers is to charge them nothing for professional services rendered them. And there are many good reasons why it should be so. As first—

1. Their means of support are usually so slender as to make it very inconvenient, and often impossible for them to pay for such services. The average salary of ministers falls short of four hundred and fifty dollars per annum. The few who reside in cities and larger villages, and who must, therefore, have larger incomes, are usually so burdened by calls of benevolence and charity, and the necessarily greater expense of living, that they are not much more able than their brethren throughout the country to pay doctor's or lawyer's bills. Secondly—

2. The life of the minister is a continual work and sacrifice of benevolence. He is expected, whether dependent upon the church or his own moderate resources, to be ever ready to do good to all men, especially to the household of faith. He is thus freely every day using learning, talents and strength for the benefit of others, which, if employed in any of the worldly professions, would secure him a competency, if not large wealth. Surely simple justice demands that such a man be dealt with most generously in return. And to mention but one other fact—

3. The medical man and the lawyer are themselves almost always the greater gainers, whatever they may do for ministers of the gospel. It is a rare case in which he does not freely confer upon them and theirs more numerous and vastly more important benefits than he ever receives from them. Regarded, then, in this point of view—as a mere business transaction—they are almost always under the greater obligations to him.

And thus do *justice, generosity* and *gratitude* all come in to establish and sustain the rule of which we are speaking.

be very careful not merely to exercise his best judgment in the case, but so to act as to hold up the hands of his brother who manifested such confidence in his honor and skill as to call him in and ask his counsel.

He will be careful, therefore, not to expose any evidence he may discover of error or deficiences in the past treatment of the case; not to say or do anything calculated to elevate himself at his brother's expense; and to let all the objections he may have to offer to the past treatment of the case, and the changes he may propose, be made only to his brother; so that nothing may appear to wound the feelings of the attending practitioner, or impair the confidence of his patient in him. In a word, he will in consultation not only bring all his knowledge and skill to the relief of the patient, but so do this as to inflict no injury upon his brother, and to contribute all he conscientiously can to hold up his hands.

Hence, also, in his general course as a professional man, he will never encourage complaints or disparaging remarks about any one of his brethren; he will never lightly pronounce an opinion against them or their practice upon the representation of others, and especially of non-professional persons; he will never seek, either by word or deed, to lower them, or injure their interests, in order to exalt himself, or increase his own business.

But not only will he avoid doing them evil, he will seek also to do them good. And, therefore, he will speak a kind word for them whenever he can; he will endeavor so to use his general influence as to promote their respectable standing in the community; and when he discovers mistakes or deficiences in any one of them, he will not blaze it out before the world, or even before the profession, but—if he think it wise and proper to say or do anything at all—he will go to the man himself, and privately, and frankly, and kindly endeavor to correct the evil.

Moreover, he will have no professional secrets whereby he may advance his own interests, to the loss or injury of the profession and his brethren. On the contrary, he is willing and desirous to communicate to the whole profession any new and better means, or methods, or any improvements, which he may have discovered or invented.

If a thing is really good—whether an article of the materia medica, or a process, or an instrument, or a new view of a subject—it ought to be made known to the profession as soon as possible. The progress of science, the honor of the profession, the interests of suffering humanity—all demand such publication. And he who can set up his own petty interests in opposition to these great objects—that man shows himself destitute of a true professional spirit. His selfishness wholly disqualifies him for a liberal pursuit. Physicians have in all ages and countries sternly set themselves, as a body and individually, against *nostrums* and *quackery* of every kind, as the bane of science, a disgrace to the profession, and a hindrance to human progress. Dentists, also, must do the same thing, if they wish to merit and obtain high professional rank.

Once more—

6. The dentist ought to *keep the lawful secrets of his patient.* All professional men—lawyers, ministers, physicians, and others—must frequently have confidential communications with those who look to them for counsel or aid, and hence they must learn much of such persons, the knowledge of which they could not otherwise have. In this way they often know that of others which their delicacy or prudence would keep secret. There are many things, personal and domestic, not wrong in themselves, but in respect to which each one should maintain an entire reserve. There are others, also, seriously involving individual character, or the peace of families, which ought not ordinarily to be divulged.

Now, in regard to all these matters—whether personal or domestic—which may in his professional practice come to the knowledge of the dentist, he ought to observe a profound silence. His own character and interests, the character of the profession, the feelings or reputation of his patient, the peace of families—all these bring him under the strongest obligations of justice and honor to say nothing of these things, not even to the companion of his bosom. No human power may extort these secrets from him, or even question him upon them, except a Court of Justice. When placed upon oath before a lawfully constituted tribunal, to tell "the truth, the whole truth, and nothing but the truth"—then, and *only then*, is he at liberty to break the seal of professional confidence, and disclose all thus required of him.

It is no excuse for the loquacious dentist to say—"Why, what harm was there in speaking of the matter? It only excited a little merriment." But, we reply—the professional man has no right to be merry or to make others merry at the expense of his patient. That gentleman, for example, may innocently have not a single natural tooth in his head; or the beautiful pearly row which adorns the mouth of that lady may be all artificial; but neither of them is willing that this fact should be a matter of jocular remark. We all have feelings on these subjects; and the professional man who trifles with these feelings—to say nothing of cases where the character of individuals or the peace of families is concerned—the professional man, we say, who trifles with these feelings is unworthy of his high, confidential calling.

About the year eighteen hundred and fifteen, during a conversation in his own office with Mr. Greenwood, then a distinguished dentist in New York, he showed me a molar tooth which he had extracted from the mouth of Washington, and also a letter from the General upon the subject of a dental operation which he wished performed. Mr. Greenwood informed me on that occasion, also, that General Washington had not a single natural tooth in his head, and that he himself had inserted a complete set for him. Now, all this—as Washington had at that time been more than fifteen years in his grave, and had then become a historical character—was quite interesting to me, and would have been to any one; and it was not at all improper for Mr. Greenwood to say what he did. It was no breach of professional confidence. But had Washington been at that time living, or had the matter involved individual character or the peace of a family, this conversation, even with a physician, as I then was, would have been wrong; and that tooth, instead of being exhibited, ought, if kept at all, to have been locked up in Mr. Greenwood's private drawer. Secondly—

II. The dentist ought to conduct himself PURELY.

We use the term *purely* here in its highest and most comprehensive moral import—to include the thoughts, the feelings, the intentions, the words, the actions; and we maintain that all these should be strictly ordered by the law of purity.

The duties belonging to this head of our subject are very many, and they often demand much reflection, care, and self-discipline, faithfully to discharge them. But their vital importance must be evident to every one who will at all seriously think upon the subject. Perhaps the most melancholy case that has ever occurred in the history of the medical profession in our country, grew out of a disregard to the great law of purity. The unhappy offender brought himself to an untimely and violent end, inflicted grievous wounds upon his profession, and opened such flood-gates of demoralizing influences as deluged the whole land for more than twelve months after his miserable death.

The dentist, like every other medical man, must be much in company with females, and be a partaker of their confidence. Let him never abuse this sacred trust. Let no thought or feeling be indulged, no language be used, which he would be ashamed to utter before his own mother or sisters;—no action be seen in him that he would not do in their presence.

There are occasionally some in the profession who, either through a defect of judgment, or something worse—perhaps a desire to be thought specially kind and agreeable for popularity-sake, or from a still more reprehensible motive—they will give into undue familiarities with females—such familiarities, we mean, as only a father, or a brother, or a very intimate friend might properly use. I have known cases of this sort in which, as any sensible, pure-minded dentist might easily have foreseen, the patient went away highly displeased and determined never to go to that man's office again; and indeed nothing but a regard to her own feelings, and a shrinking back from what would injure his character and business, and mortify and disgrace his family, prevented her from exposing his conduct.

We are far from supposing that a female should always be accompanied by a friend when she goes to have an operation performed upon her teeth. The strictest regard to propriety requires no such rule. But we do think that the circumstances are very rare which will justify a dentist in administering chloroform or any of the anæsthetic agents to a female without an attendant, or, at least, some third person present. What a fearful amount of suffering and loss would that unhappy man in one of our eastern cities have saved himself and family, had he only observed this rule! It would not then have been left to subsequent providential developments to show his probable innocence and deliver him from an ignominious imprisonment.

Be assured that perfect purity of conduct—to say nothing of duty—perfect purity of conduct is always the best *policy.* Let the dentist, then, while careful to be ever kind and accessible—let him always conduct himself with such marked purity of spirit and manners as in this way to draw, as it were, a circle, a sacred circle, about himself, beyond which nothing shall ever tempt him to go, and through which the most thoughtless or loose will not dare to break. This is his only safety; it will prevent others from a dangerous familiarity with him, and keep him, also, from undue liberties with them. And all this the professional man can be and do without rendering himself, in the least degree, a cold, unfriendly, repulsive man.

III. Let the dentist practice his profession BENEVOLENTLY.

A man may be just and pure, and yet lack benevolence.

Now, such a man we must respect, but we can scarcely love him. There is little in him to attract us; we can not look upon him with delight. Take away the beautiful tints and the delicious fragrance of the rose, and you rob it of all that is sweet and delightful. Its charms are gone. So benevolence is the beauty, the aroma of a good character; it gives to it its most enchanting qualities. Our consciences will indeed calmly bear a testimony for the upright and the pure; but our bosom's warmest affections spontaneously flow out towards the benevolent man. He, then, who would attain to the highest style of character must cultivate the virtue of benevolence. He who wants this excellence falls miserably short of the true professional man.

Let it be here repeated that the very spirit of every profession, as distinguished from a mere money-making trade or art, is benevolence. This is its true glory. Hence, no profession, unless its practitioners are manifestly men of a benevolent character, can long command the respect of the community, or rapidly advance in the career of improvement. The dentist who has not this spirit is utterly unfit for the position and the duties of his liberal calling.

Further, it is good policy, also, in the professional man to be benevolent; for the public will be sure, sooner or later, to find him out and bestow their richest favors upon him. The people delight to honor such a man.

But, my young friends, God has not left this important element of character to rest merely upon what is becoming a liberal profession, or tends to its advancement, however noble these considerations may be; neither has He based its practice solely upon views of policy or individual interests. No; benevolence comes to us with all the obligations of duty; it is, strictly speaking, a duty towards our fellow-men. O, there is a world of meaning and of obligation, too, in that simple testimony of Holy Scripture, "*The poor ye have always with you.*" And does not earth's whole history bear witness to this inspired declaration? The cries of suffering humanity are continually going up to Heaven, and calling down direst maledictions upon the ear and the heart that can shut out these wailings. It was a noble saying of Boerhaave, "*The poor are my best patients, for God is their paymaster.*"

One more thought on this point. No class of men can do so much good at so little expense as the medical man, whether he be physician, surgeon, or dentist. A slight operation, or a cheap prescription, or even a word or two of advice, may speedily remove a disease which otherwise might cause months of suffering and perhaps death; and often restore to his labors the poor man whose half-starved wife and little ones are dependent upon his efforts for their daily bread. What an amount of misery is thus prevented! What an amount of relief is thus conferred!

And my position being such that my testimony can not be suspected, I may here be permitted to add, "I have been young and now am I old, yet never saw I" the men or class of men engaged in this world's callings who exhibited so much of the spirit and the practice of a true benevolence as medical men.

It will be noticed, my friends, that in all we have now said, we have associated the conduct with the heart, the act with the principle. If, for example, the dental practitioner is required to be faithful in his work and equitable in his charges, it is because justice demands it. If required that he should avoid all impure language and conduct and undue familiarity with his female patients, it is because a pure heart exacts such a deportment. Just as benevolence in the soul also requires that he should compassionate the poor and be kind toward all men.

Is it not manifestly the right feeling in the bosom that gives value to all our doings? The latter separated from the former are mere appearance—put on to serve some selfish purpose. There is no true virtue in such conduct. Moreover, it can not be depended upon in an emergency; it can not endure thorough trial; and at the best it is quite imperfect. Any one of moderate discernment can usually look through a mere appearance of virtue; and even should such a man escape the discovery and condemnation of his fellow men, the day is coming when the mask will be torn away and the hypocrite will stand revealed in his true character.

And this leads us here to offer a few remarks upon the true and only foundation, and standard of all morality. What are these? *A reverential regard to the authority of God deeply implanted in the heart, is the sole basis of virtue.; and his perfect law, as revealed in his word, is our supreme directory.* Without these we are all afloat upon a dark and agitated sea of conflicting opinions, interests, and passions, and will be sure, sooner or later, to make shipwreck of character and happiness. God's throne is eternal; every thing else is changeable: "all his commandments are sure. They stand fast for ever and ever."

We do not mean, indeed, that a virtuous man can not or ought not to be influenced by other motives than a regard to the authority and the law of God. His temporal interests are a proper subject of consideration, and it would be culpable in him to overlook these; and he is bound, also, to conform, so far as he innocently can, to what is considered becoming in society, and not needlessly to violate any of its proprieties; and he ought also to seek, in every legitimate way, to please his fellow men. But all these things, however important in themselves, are to be held in subordination to the authority and the law of God. Hence, the instant they come into conflict with the divine rule, and the divine standard—all these, our temporal interests, the opinions and customs of society, the feelings and wishes of our fellow men—all these must be at once given up. God must be obeyed at all hazards, and whatever be the sacrifice. He who does not do this is not a truly moral man. And he who does do this, will be sure in the long run to be the greatest gainer, because he himself, and the whole system of things, have been so formed and adjusted, that virtue is always, and in every case, our true interest. However the good man may suffer for a while, he must finally triumph, so sure as God is on the throne. And now does not this perfect harmony between our nature and the system of things, on the one hand, and the moral law on the other, prove that these all proceeded from the same divine author? Verily, He who made us and the world around us, made the Bible also. These are all in their constitution and legitimate workings, perfectly adjusted to each other. They manifestly proceeded from the same divine hand.

But, further, to any one who will carefully examine his own heart and life, and compare these with the law of God, it will be manifest that he has often fallen short of the divine standard—often left undone what he ought to have done, and often done what he ought not to have done. In a word, his conscience, honestly consulted, his own conscience, will convict him of sin. And he will be sensible, too, if at all seriously reflecting, that his sins have all been unspeakably aggravated by the "riches of God's goodness, forbearance, and long-suffering," continually manifested toward him.

Hence, these two questions will press upon that man's attention with a solemn weight—how can I obtain the pardon of my past offences? And how can I be guided and sustained in the path of duty hereafter? And he who does not wilfully close his eyes to these great questions, or seek to drown the voice of conscience by plunging more deeply into the cares or pleasures of the world, that man will sooner or later, find that the GOSPEL, and the Gospel alone, discloses a remedy for the guilt, the ignorance, and the moral weakness of the sinner. In the mediation of the divine Redeemer he will see the law of God, which we have all broken, magnified and made honorable, the pillars of the divine government upheld in all their strength and grandeur, while a holy pathway for mercy is opened to a guilty world. "God was in Christ reconciling the world unto himself."

And blessed be the giver of all good and perfect gifts—

we are not bid to wait until the day of judgment, or till we enter the future state, for an experience of the divine mercy. Even this would indeed be great goodness: but the Gospel offer is vastly more; it offers us a *present*, an immediate *salvation.* God will save us *now.* Yes, if we simply come in faith to the Cross of Christ, we shall there find the double blessing—*pardon* and a *renewal into holiness*—that blessing of which we all stand in perishing need. Peace with God, and a pure heart will at once be ours. So that we shall not only experience a relief from all our guilty fears, but we shall, through grace, become altogether changed in the spirit of our mind, our whole character and walk. "If any man be in Christ," declares the Apostle, "he is a new creature; old things are passed away: behold all things are become new." (2 Cor. v. 17.) Speaking of this change, one of the greatest intellects the world ever saw—a man who lived in the fourth century—we mean Augustine—and who was himself a most distinguished example of the truth of what he testified;—"This *change*," says Augustine, is "*a greater work than to make such a new heaven and earth as is already made.*"

But when God, our Savior, promises in the Gospel to give *now* and to *all* who sincerely seek these blessings—light on the subject of their great interest, and a true peace, and a new and holy heart, so that they will be enabled in the time to come, to triumph over temptation, and cheerfully to do God's will in all things; when, we say, the divine Savior promises to do all this *here* and *now* in the case of each and every one who truly comes to him;—see ye not, my beloved friends, that in this very offer He gives to us all the means and the opportunity of at once testing his veracity? Yes—O the wonderful condescension of infinite Truth and Love! God, our Savior, gives to each and to all of us *now* the means and the opportunity of at once testing his veracity—of knowing for ourselves the truth of his promises.

And thousands and tens of thousands, in every age, have thus taken the Savior at his word, and found it, in their own joyful experience, to be fulfilled. They themselves have become conscious in themselves of this gracious change of state and disposition; and the world around them, too, has seen most convincing evidence of the accomplishment of this wonderful promise in their new and holy character, and in their just, pure, and benevolent conduct.

No one, be assured, ever really sought this great blessing without finding it. And no one, till he has thus faithfully sought it, and then (were this possible) been disappointed in his earnest expectations and endeavors, has any reason or right to call in question the experience of others, or to indulge a single doubt of the promises of the Gospel, or of the reality of that great spiritual and moral transformation which the glorious Gospel of the ever blessed God engages, under the power of the Holy Spirit, to work now in all them who truly come to the Savior.

And now, my young friends, if you have not already found these wonderful and infinitely precious blessings of the Gospel, seek them early! we entreat you—seek them faithfully. Yours will then be, through the riches of divine grace, that peace which the world can neither give nor take away, even peace with God, through Jesus Christ, our Lord. And yours will also then be that just, pure, benevolent character by which alone you can be fitted for usefulness and happiness in this life, and the higher usefulness and happiness of the life to come.

"O thou bleeding Love!
The grand morality is love of THEE!"—YOUNG.

